# Exploring socio-economic inequalities in mental healthcare utilization in adults with self-reported psychological distress: a survey-registry linked cohort design

**DOI:** 10.1017/S2045796024000842

**Published:** 2025-01-23

**Authors:** J. J. Muwonge, C. Dalman, B. Burström, B. Jablonska, A-C. Hollander

**Affiliations:** 1 Department of Global Public Health, Karolinska Institute, Stockholm, Sweden; 2 Centre for Epidemiology and Community Medicine, Stockholm Health Care Services, Region Stockholm, Stockholm, Sweden

**Keywords:** inequities, covid-19, psychiatric services, Sweden, GHQ-12, kessler six, digital services, psychotherapy, primary care, specialized care

## Abstract

**Aims:**

Although individuals with lower socio-economic position (SEP) have a higher prevalence of mental health problems than others, there is no conclusive evidence on whether mental healthcare (MHC) is provided equitably. We investigated inequalities in MHC use among adults in Stockholm County (Sweden), and whether inequalities were moderated by self-reported psychological distress.

**Methods:**

MHC use was examined in 31,433 individuals aged 18–64 years over a 6-month follow-up period, after responding to the General Health Questionnaire-12 (GHQ-12) in 2014 or the Kessler Six (K6) in 2021. Information on their MHC use and SEP indicators, education, and household income, were sourced from administrative registries. Logistic and negative binomial regression analyses were used to estimate inequalities in gained MHC access and frequency of outpatient visits, with psychological distress as a moderating variable.

**Results:**

Individuals with lower education or income levels were more likely to gain access to MHC than those with high SEP, irrespective of distress levels. Education-related differences in gained MHC access diminished with increasing distress, from a 74% higher likelihood when reporting no distress (odds ratio, OR = 1.74 [95% confidence interval, 95% CI: 1.43–2.12]) to 30% when reporting severe distress (OR = 1.30 [0.98–1.72]). Comparable results were found for secondary care but not primary care i.e., lower education predicted reduced access to primary care in moderate-to-severe distress groups (e.g., OR = 0.63 [0.45–0.90]), and for physical but not digital services. Income-related differences in gained MHC access remained stable or increased with distress, especially for secondary care and physical services.

Among MHC users, we found marginal socio-economic differences in the frequency of outpatient visits, and these differences decreased with increasing distress. Yet, having only primary education with severe distress was associated with fewer outpatient visits compared with having post-secondary education (rate ratio, RR = 0.82; 95% CI: 0.67–1.00). These inequities were especially evident among women and for visits to psychologists, counsellors, or psychotherapists.

Although lower-income groups used services more than others, they still had higher odds of not using services when reporting distress (i.e., those not in contact with services despite scoring ≥3 on the GHQ-12 or ≥8 on the K6; OR = 1.27; 95% CI: 1.15–1.40).

**Conclusions:**

Overall, individuals with lower education and income used MHC services more than their counterparts with higher socio-economic status; however, low-educated individuals faced inequities in primary care and underutilized non-physician services such as visits to psychologists.

## Background

While a clear social gradient exists in poor mental health, with lower socio-economic position (SEP) linked to a higher burden of mental disorders (Kivimäki *et al.*, 2020), the utilization of mental healthcare (MHC) does not necessarily follow this gradient. In settings where MHC is mostly paid by the user, either out of pocket, or with insurance schemes, the common finding is that individuals with lower SEP utilize less MHC than their affluent peers (Bartram, 2019). In settings with publicly financed healthcare, studies have reported both less (Butterworth *et al.*, 2013; Epping *et al.*, 2017; Geyti *et al.*, 2020; Leppänen *et al.*, 2022; Packness *et al.*, 2021) and more utilization of MHC by individuals with lower SEP compared with their peers (Butterworth *et al.*, 2013; Epping *et al.*, 2017; Geyti *et al.*, 2020; Gudmundsdottir and vilhjalmsson, 2010; Jablonska *et al.*, 2016; Loef *et al.*, 2021; Paananen *et al.*, 2013; Walker *et al.*, 2019). However, it is difficult to ascertain if the observed inequalities in MHC use are due to differences in need rather than socio-economic status, as only a few studies have accounted for need for MHC (Butterworth *et al.*, 2013; Geyti *et al.*, 2020; Gudmundsdottir and vilhjalmsson, 2010; Loef *et al.*, 2021; Packness *et al.*, 2021).

Access to MHC is an effective way to treat mental disorders (Ghio *et al.*, 2015), and promoting equity in MHC – offering equal care for the same level of needs (horizontal equity) or more care for greater needs to ensure similar outcomes (vertical equity) – could reduce the excess poor health in individuals with lower SEP (Ghio *et al.*, 2015; Gulliford *et al.*, 2002). Equitable healthcare is the cornerstone of the Swedish Health and Medical Service Act (Sveriges-riksdag, 2017). In addition, the out of pocket expenditure is limited, with adults charged a user fee of 275 Swedish crowns (in Stockholm County since 2024) per visit and a 12-month ceiling of 1400 Swedish crowns (Region-stockholm, 2024).

An ecological study comparing psychological distress at the group level with registry-recorded MHC utilization found potential unmet needs among residents in less affluent areas of Stockholm (Jablonska *et al.*, 2021). However, as the study had an ecological design it may be associated with the so called ecological fallacy, that is, associations observed at the group level are not always present at the individual level (Glynn and wakefield, 2010). This study aims to investigate socio-economic inequalities in MHC use among individuals with varying levels of self-reported psychological distress. We hypothesize that if inequalities in MHC use exist, they should reduce and become negligible with increasing severity of reported distress (need).

## Methods

### Setting

This study was conducted in Stockholm County, which is administered by Region Stockholm and is the most populous of the 21 counties in Sweden. MHC is primarily provided by region-financed private and public facilities. The region has a system of stepped care, contingent on assessed needs, starting with primary care, and if needed, adult psychiatry (specialized outpatient and inpatient care including compulsory psychiatric care), with emergency departments handling acute cases.

### Materials/data sources

The Stockholm Public Health Cohort (SPHC), owned by Region Stockholm, is a survey-registry linkage database. It includes a cohort and data on a random cross-sectional sample of Stockholm residents (aged 16+) who participated in the *Hälsa Stockholm* surveys between 2002 and 2021. Among other questions, participants responded to the General Health Questionnaire (GHQ-12; in all survey years except 2021) and the 6-item Kessler psychological distress scale (K6; only in 2021). For this study, the two most recent waves, 2014 and 2021, were pooled. With a few exceptions, the sampling and data collection procedures were consistent across the waves. The response rate was higher in the 2021 wave (48.2%) compared with the 2014 wave (42.3%). The samples were relatively comparable in terms of sociodemographic factors. However, more individuals used MHC in 2021 than in 2014 (*see page 1 of the appendix for details on pooling).*

MHC use was collected from the regional healthcare database, ‘*VAL-databaserna’*, which contains information on prescribed drugs and healthcare records in public and region-financed private facilities in Stockholm County (Svensson *et al.*, 2012). Additional data on prescribed drugs were collected from the National Prescribed Drugs’ Registry. Education status and household income were collected in the same year as the surveys from the longitudinal integration database for Health Insurance and Labor Market Studies and the total population registry (TPR) from Statistics Sweden (SCB).

### Study design

This study employed a survey-registry linked cohort design to examine equity in MHC use among adults. MHC use was measured over a 6-month period from the date of participants’ survey responses regarding psychological distress. A 6-month follow-up period was selected to capture MHC use close to the period of distress.

### Study population

A random sample of 45,316 individuals, aged 16 years and older, participated in 2014 or 2021. After the exclusion of three groups: adolescents aged 16–17, individuals aged 65 and above, and those who had died or emigrated during the 6-month follow-up period, our final sample consisted of 31,433 individuals (Fig. S1).

### Study variables

#### MHC use

MHC use was defined as outpatient visits or inpatient admissions within primary and secondary care if a participant (1) had a recorded psychiatric diagnosis, (2) met mental health professionals or (3) collected psychotropic medication (*see page 1 of the appendix for additional details including ICD and ATC codes*).

MHC use was grouped into the following:

1. Gained access to MHC services indicated by MHC use at least once during 6 months of follow-up after survey-response. We analyzed total MHC access, by healthcare level, and type of visit.
2. Continuation/progression in care indicated as the frequency of outpatient visits among those with MHC access during the 6-month follow-up. We analyzed total outpatient visits, and by provider met i.e., (a) general practitioner (GP) or psychiatrist and (b) psychologists, counsellors, or psychotherapists.

#### Indicators of SEP

Education status and household income were selected to indicate an individual’s social position in society and access to necessary resources important for MHC use (Patel *et al.*, 2018; Whitehead *et al.*, 2016).

- Education status was categorized into the following: ‘primary’, ‘secondary’, and ‘post-secondary’.
- Equivalized disposable household income is calculated by SCB for each household/year and is weighted for household size. Participants’ disposable income was categorized into tertiles: ‘Low’, ‘Middle’, and ‘High’.

#### Self-reported psychological distress

Psychological distress, measured using the GHQ-12 in 2014 and K6 in 2021, was used as an indicator of MHC needs. Both the GHQ-12 and K6 are validated instruments that are commonly used to screen for psychological distress in population-based surveys (Lundin *et al.*, 2017; Prochaska *et al.*, 2012). Both measure how often or how much symptoms have affected a person’s functionality in the past few weeks (GHQ-12) or month (K6) (Kessler *et al.*, 2002; Lundin *et al.*, 2017). The GHQ-12 consists of a score ranging from 0 to 12 (bi-modal scoring 0 0 1 1) while the K6 has a total score range from 0 to 24 (0 1 2 3 4), with higher scores representing increasing severity of distress (Lundin *et al.*, 2017; Prochaska *et al.*, 2012).

Individuals were grouped into three categories based on their scores on the GHQ-12 or K6: ‘no distress’, ‘moderate distress’ and ‘severe distress’ (Table 1), following prior K6 studies (Folkhälsomyndigheten, 2021; Prochaska *et al.*, 2012) and a conversion table from a study equating the GHQ-12 and K6 (Lundin *et al.*, 2024).

**Table 1.** Creation of moderator levels using scores from the K6 and GHQ-12

	No distress	Moderate distress	Severe distress
K6	0–4	5–12	13–24
GHQ-12	0	1–7	8–12

#### Covariates

- Age was used as a continuous variable in analyses and categorized into 18–29 and 30–64 for age-stratified analyses.
- Sex assigned at birth was collected from the TPR already categorized into ‘Men’ and ‘Women’.
- Migration status was categorized into four mutually exclusive categories: born in ‘Sweden’, ‘Nordic, other’, ‘Europe, other’ and ‘outside Europe’.
- Survey year was either ‘2014’ or ‘2021’ and used to adjust for survey-wave differences.

### Statistical analysis

A logistic regression analysis for complex surveys was performed to estimate the association between SEP and MHC use at least once during follow-up. Effect modification by distress was examined using interaction analyses, with results presented across distress strata to visualize changes with increasing distress severity. Both crude and adjusted odds ratios (OR) with 95% confidence intervals (CIs) are shown within each distress stratum, along with *p*-values from Wald tests to show the significance of observed differences across distress strata.

A negative binomial regression for complex surveys was performed to estimate the association between SEP and the frequency of visits in outpatient services among MHC users. Estimates are presented across distress strata, with crude and adjusted rate ratios (RRs) and 95% CIs, including Wald test *p*-values.

Total, sex- and age-stratified analyses were performed, with age, sex, survey year and migration status included in the adjusted models. In addition, survey (calibration) weights were applied in each analysis to reduce nonresponse bias and improve external validity.

### Handling of missing values

Complete case analysis was performed due to a low proportion of missing (<1.2%).

### Sensitivity analysis & post hoc analysis

Because individuals with previous long term sickness absence might have lower SEP and higher service use, we excluded those with at least 56 days of sickness absence (cut-off used previously [Karlsson *et al.*, 2008]) the year they responded to the surveys (*n* = 1142). The results (not shown) were similar to the main findings. In addition, sensitivity analyses excluding participants who used MHC 6 months prior to the surveys (*n* = 5379) was performed to remove the effects of previous use on reporting of distress and use of services during the 6-month follow-up period.

Additional analyses were performed to examine socio-economic inequalities in not using services during the 6-months’ follow-up, despite scoring ≥3 on the GHQ-12 or ≥8 on the K6.

All analyses were performed in STATA version 17, with graphs produced in R-studio (R version 4.2.2) (*see page 2 of the appendix for the STATA syntax*).

## Results

We studied 31,433 adults aged 18–64 (mean age = 43.6 years), majority of whom were women (54.9%). Most were born in Sweden (78.6%) and had higher SEP (Table 2).

**Table 2.** The characteristics of the study sample by reported psychological distress. Unweighted frequencies and percentages presented

	Total		No distress		Moderate		Severe		Missing	
**Characteristics**	***N* (%)**	**%MHC use^+^**	***n* (%)**	**%MHC use^+^**	***n* (%)**	**%MHC use^+^**	***n* (%)**	**%MHC use^+^**	***n* (%)**	**%MHC use^+^**
**Total**	**31 433 (100%)**	**18.1%**	**17 722 (100%)**	**10.9%**	**10 862 (100%)**	**22.9%**	**2486 (100%)**	**47.2%**	**363 (100%)**	**22.6%**
**Sex**										
Men	14 179 (45.1%)	12.7%	8810 (49.7%)	8.0%	4318 (39.8%)	17.2%	868 (34.9%)	36.9%	183 (50.4%)	20.2%
Women	17 254 (54.9%)	22.5%	8912 (50.3%)	13.9%	6544 (60.2%)	26.6%	1618 (65.1%)	52.7%	180 (49.6%)	25.0%
**Age**										
18–29	5 337 (17.0%)	17.6%	2142 (12.1%)	10.1%	2471 (22.7%)	17.3%	678 (27.3%)	42.2%	46 (12.7%)	15.2%
30–64	26 096 (83%)	18.2%	15580 (87.9%)	11.1%	8391 (77.3%)	24.5%	1808 (72.7%)	49.1%	317 (87.3%)	23.7%
**Survey wave**										
2014	15 490 (49.3%)	16.7%	8624 (48.7%)	10.9%	5607 (51.6%)	19.8%	1172 (47.1%)	44.5%	87 (24.0%)	18.4%
2021	15 943 (50.7%)	19.4%	9098 (51.3%)	11.0%	5255 (48.4%)	26.1%	1314 (52.9%)	49.6%	276 (76.0%)	23.9%
**Education level**										
Primary	2 698 (8.6%)	23.5%	1332 (7.5%)	14.0%	973 (9.0%)	27.4%	330 (13.3%)	50.3%	63 (17.4%)	22.2%
Secondary	10 811 (34.4%)	18.3%	6113 (34.5%)	10.9%	3629 (33.4%)	22.4%	924 (37.2%)	49.1%	145 (39.9%)	28.3%
Post-secondary	17 663 (56.2%)	17.1%	10115 (57.1%)	10.4%	6196 (57.0%)	22.4%	1207 (48.6%)	45.1%	145 (39.9%)	17.2%
Missing	261 (0.8%)	20.7%	162 (0.9%)	18.5%	64 (0.6%)	20.3%	25 (1.0%)	36.0%	10 (2.8%)	20.0%
**Household income**										
Low	8 947 (28.7%)	24.2%	4113 (23.2%)	14.2%	3542 (32.6%)	26.7%	1106 (44.5%)	53.1%	186 (51.2%)	27.4%
Middle	11 255 (35.8%)	16.8%	6368 (35.9%)	9.9%	3969 (36.5%)	21.8%	824 (33.1%)	46.0%	94 (25.9%)	22.3%
High	11 220 (35.7%)	14.4%	7232 (40.8%)	10.0%	3349 (30.8%)	20.1%	556 (22.4%)	37.2%	83 (22.9%)	12.0%
Missing	11 (0.0%)		2 (0.0%)		3 (0.0%)					
**Migration status (birth country)**										
Outside Europe	3 812 (12.3%)	14.6%	1888 (10.7%)	7.6%	1386 (12.8%)	17.0%	408 (16.4%)	38.0%	130 (35.8%)	16.9%
Europe	2 112 (6.7%)	14.8%	1159 (6.5%)	8.5%	717 (6.6%)	16.2%	183 (7.4%)	47.5%	53 (14.6%)	20.8%
Nordic, other	811 (2.6%)	20.6%	494 (2.8%)	13.2%	245 (2.3%)	27.8%	60 (2.4%)	51.7%	12 (3.3%)	25.0%
Sweden	24 695 (78.6%)	18.8%	14180 (80.0%)	11.5%	8512 (78.4%)	24.2%	1835 (73.8%)	49.0%	168 (46.3%)	27.4%
Missing	3 (0.0%)		1 (0.0%)		2 (0.0%)		–		–	

(+) %MHC use is a row percentage e.g., the proportion of the ‘Total’ number of men who used any MHC is 12.7%, while among men who reported ‘No distress’, the proportion who used any MHC is 8.0%. The corresponding proportion who used any MHC among those who reported ‘Moderate distress’ and ‘Severe distress’ is 17.2% and 36.9%, respectively.

Similarly, among people born in other Nordic countries, the total proportion who used any MHC is 20.6%, this proportion increases to 51.7% if they reported severe distress.

Over half reported no distress (56.4%), about 35% (weighted prevalence = 36.2%) reported moderate distress, and 7.9% (weighted prevalence = 8.6%) reported severe distress. Those reporting moderate to severe distress were more likely women, younger (18–29), with lower SEP, and born outside the Nordic region (Table 2).

Individuals with moderate-to-severe distress were more likely to use MHC than those with no distress. For instance, 47.2% with severe distress used MHC, compared with 10.9% with no distress. At all levels of distress, MHC use was highest among women, those aged 30–64, in the 2021 wave, participants born in other Nordic countries, and those with the lowest SEP (Table 2).

### Distress-moderated differences in gained MHC access

#### Education-related differences

**Figure 1.**
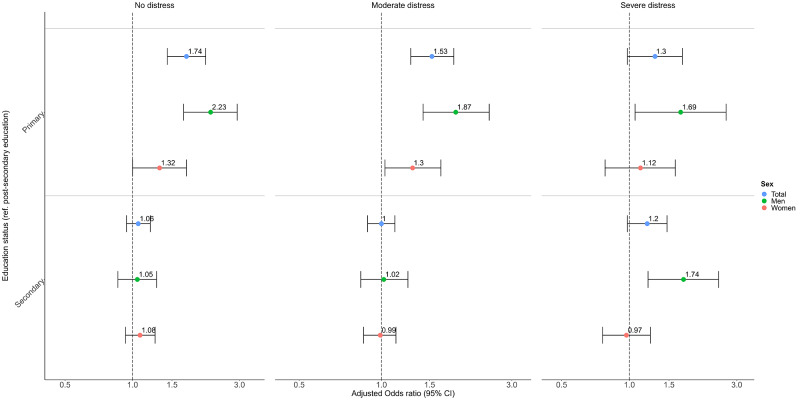
Odds ratios for the moderated association between education status and any MHC use at least once within 6 months after survey response.

Individuals with only primary education were more likely to gain access to MHC services than those with post-secondary education. These differences diminished with increasing distress, from a 74% higher likelihood when reporting no distress (OR = 1.74 [95% CI: 1.43–2.12]) to 30% when reporting severe distress (OR = 1.30 [0.98–1.72]), though not significantly (Wald test, *p* = 0.2414; Fig. 1 & Table S2). Differences in gained MHC access between individuals with secondary education and those with post-secondary education were not significant at any distress level (Fig. 1 & Table S1).

Education-related differences in gained MHC access were larger in men than in women. For men, differences in gained MHC access between those with secondary education and those with post-secondary education widened with increasing distress (Wald test, *p* = 0.0310; Fig. 1 & Table S2). Younger adults showed larger education-related differences compared with older adults (Table S3).

Patterns varied by healthcare level and visit type. Lower education status was linked to reduced odds of accessing primary care, particularly among individuals with moderate-to-severe distress (e.g., OR = 0.63 [0.45–0.90] primary vs post-secondary education with severe distress), but increased odds for accessing secondary care (OR = 2.10 [1.53–2.87] primary vs post-secondary education; Fig. S2 & Table S4). Lower education status was also linked to increased odds of accessing physical outpatient services (e.g., OR = 1.42 [1.06–1.89] primary vs post-secondary education) but not digital services (OR = 1.12 [0.77–1.61] primary vs post-secondary education; Fig S4 & Table S5).

#### Income-related differences

**Figure 2.**
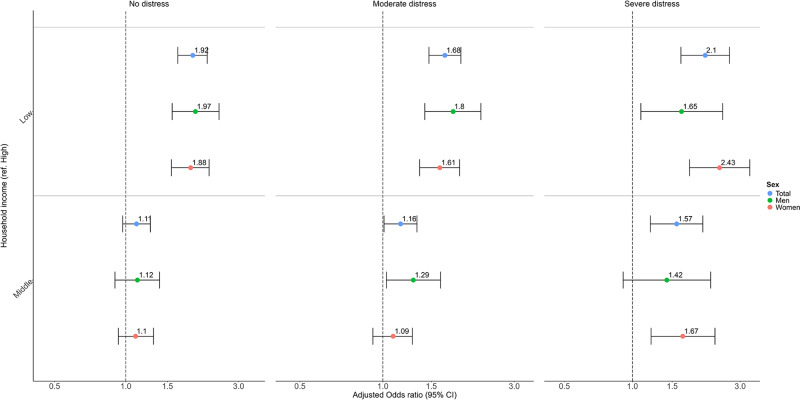
Odds ratios for the moderated association between household income and any MHC use at least once within 6 months after survey response.

Individuals with lower household income were more likely to gain access to MHC services than those with higher income in all distress strata (Wald test, *p* = 0.1914; Fig. 2 & Table S1). Differences in gained MHC access between middle-income and higher-income groups widened with increasing distress. The OR increased from 1.11 (95% CI: 0.97–1.27) in those with no distress to 1.57 (1.20–2.05) among those with severe distress (Wald test, *p* = 0.0697). Sex and age-stratified analyses showed a pattern of results comparable to the main findings above (Fig. 2 & Tables S2–S3). In addition, income-related differences in MHC access were present in both primary and secondary care, and by type of visit but differences were much larger in secondary care and for physical visits (Table S4–S5).

#### Results from the sensitivity analysis

Excluding participants who used MHC services within 6 months prior to survey participation returned mostly attenuated and non-significant education-related differences for those with moderate-to-severe distress. Income-related differences were also attenuated but remained significantly high especially for individuals with severe distress (Table S6).

### Distress-moderated differences in the frequency of outpatient visits among MHC users (N = 5676)

#### Education-related differences

**Figure 3.**
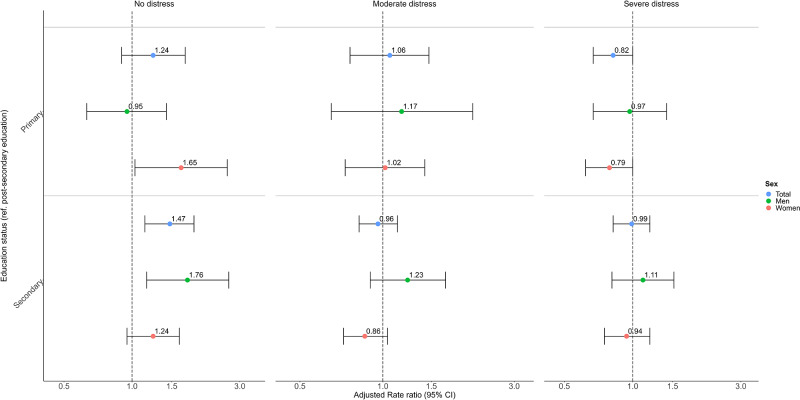
Rate ratios for the moderated association between education status and the frequency of outpatient visits within 6 months after survey response among MHC users.

Among MHC users, we generally found no significant education-related differences in the frequency of outpatient visits (Fig. 3). RRs were higher in the group reporting no distress and reduced to around one in those with moderate-to-severe distress (Wald test, *p* = 0.0771 primary education & *p* = 0.0170 for secondary education; Fig. 3 & Table S7). However, among those with severe distress, individuals with only primary education visited services less frequently than those with post-secondary education (RR = 0.82; 95% CI: 0.67–1.00; Fig. 3 & Table S7). Sex-stratified analyses showed similar results in women (RR = 0.79 [95% CI: 0.62–1.00]) but not in men (RR = 0.97 [95% CI: 0.67–1.40]; Fig. 3 & Table S8). Age-stratified analyses showed no clear difference from the main results, except that younger adults with secondary education had fewer visits than their peers with post-secondary education in the severe distress group (Table S9).

There were mostly no significant education-related differences in visits by type of provider. However, low-educated individuals tended to visit psychologists, counsellors, and psychotherapists less frequently, particularly when reporting moderate-to-severe distress (Fig. S7 & Table S10).

#### Income-related differences

**Figure 4.**
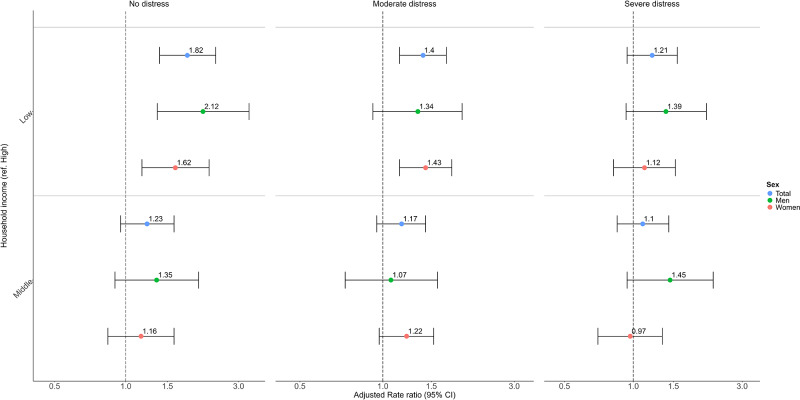
Rate ratios for the moderated association between household income and the frequency of outpatient visits after survey response among MHC users.

Low-income individuals visited outpatient services more frequently than high-income individuals (Table S8), but differences diminished with increasing distress. For instance, the RR decreased from 1.82 (95% CI: 1.39–2.40) when reporting no distress to 1.21 (95% CI: 0.94–1.56) when reporting severe distress (Wald test, *p* = 0.0965; Fig. 4 & Table S7). Comparisons between middle- and high-income individuals produced marginal and non-significant differences (Table S7). Sex-stratified analyses showed similar results as the main results (Fig. 4 & Table S8). Age-stratified analyses showed similar results among older adults, but not for younger adults, income-related differences in visit frequency increased with severity of distress (Table S9). Analyses by type of provider showed significant income-related differences in visits to GPs/psychiatrists but not to other mental health providers (Table S10).

### Not using services despite high levels of distress (≥3 on the GHQ-12 or ≥8 on the K6)

After adjusting for age, sex, migration status and survey year, lower income groups were 18–27% more likely than their higher-income counterparts not to use MHC services despite reporting high levels of distress. The ORs were 1.27 (95% CI: 1.15–1.40) for the lowest versus highest income group and 1.18 (95% CI: 1.07–1.29) for the middle versus highest income group. Education-based differences were only significant in women and older adults (Table S11).

## Discussion

This survey-registry linked study aimed to explore inequalities in MHC use among adults while considering their self-reported psychological distress (proxy for MHC needs). Individuals with lower SEP had higher MHC needs and used MHC more than their affluent peers.

Among service users, those with lower SEP had more frequent visits than their affluent counterparts but differences were minor among patients with moderate-to-severe distress, suggesting an equitable distribution of visits. Nonetheless, among patients with the most need for services, low-educated patients underutilized non-physician services.

### Comparison to previous studies and explanation of our findings

#### Gained access to services

Our finding that individuals of lower SEP utilize more MHC than their affluent counterparts aligns with some studies in countries with strong universal healthcare systems (Annequin *et al.*, 2015; Crump *et al.*, 2011; Jokela *et al.*, 2013; Packness *et al.*, 2017). This higher utilization among individuals with lower SEP is positive, given that they experience more mental health problems and a greater impact from illness (Bartram, 2019). While this suggests that material conditions like income may not influence access to MHC services in Sweden, where financial barriers are minimal, an alternative explanation could be social selection, where existing illness leads to further socio-economic deprivation (Mossakowski, 2014). However, our sensitivity analysis, excluding individuals with long-term sickness absence, returned similar results, suggesting the findings are consistent.

Horizontal equity, defined as equal care for similar need regardless of SEP, was examined. Differences in gained MHC access between groups were only slightly attenuated with increasing level of distress, suggesting ‘inequalities’ in favour of the less affluent. However, higher MHC use by individuals with lower SEP ‘cannot be interpreted as unfair’, given their higher underlying need for services and higher unmet needs (Wagstaff *et al.*, 2007; Whitehead and dahlgren, 2006). In other words, their higher use of MHC services may still be insufficient in proportion to their level of need, at least at the population level.

#### Frequency of service utilization

Our study found a more equitable pattern of outpatient service utilization (with increasing severity of distress) among individuals who had contacted care, regardless of their SEP, reflecting horizontal equity. Nevertheless, there were exceptions to this finding.

Among those with the greatest needs, persons with only basic education had fewer visits to psychologists, counsellors, or psychotherapists. This lower utilization, also observed in other studies (Halme *et al.*, 2023; Leppänen *et al.*, 2022; Packness *et al.*, 2017), may be due to highly educated people preferring psychotherapy, which is perceived as less stigmatizing (Backenstrass *et al.*, 2006). Mental health literacy, time constraints, costs and severity of symptoms at the time of seeking services could further explain these disparities in psychotherapy or counselling (Holman, 2015).

#### Differences by healthcare level and type of visits

Individuals with lower education status had lower rates of primary care visits but higher rates of specialized visits than their peers. This may suggest delayed help-seeking, leading to more severe problems that are typically managed in secondary care.

Inequalities in the use of digital services were minimal or non-significant. Previous studies indicate that digital services in Sweden are used predominantly by younger, native-born, highly educated individuals in urban areas (Wilkens *et al.*, 2024). During the covid-19 pandemic, physical services were restricted and digital services were promoted to enhance access (Ohlis *et al.*, 2020). As digital technologies become increasingly integrated into traditional MHC services, monitoring equity in access remains crucial (Kalman *et al.*, 2023).

### Strengths and limitations

By linking population-based surveys and high-quality registry data, we accessed information on self-reported psychological distress, an indicator of subjective need for MHC, and subsequent MHC use from registries, minimizing recall bias. The large sample size allowed us to detect small differences by SEP across groups and by healthcare level, visit type, and provider.

Potential limitations include selection bias, as survey responders may differ from the target population. For instance, if less affluent individuals with high baseline MHC use were more inclined to participate, then we might have overstated the inequalities. However, a study by Agerholm *et al.* (2016) found comparable socio-economic inequalities in healthcare use among survey responders and the total population. Additionally, response rates below 50% may mean that we fail to capture inequities experienced by the most vulnerable in society, such as those with severe mental illness, who are less likely to respond to surveys.

Although self-reported psychological distress is a significant predictor of care seeking (Roberts *et al.*, 2018; Sun *et al.*, 2018), it might not fully capture an individual’s MHC needs. Some individuals may experience temporary distress that resolves without treatment, while others may require treatment despite not reporting distress. Nevertheless, in population-based surveys, where costs are high, self-administered distress scales provide a practical way to identify probable needs, which can then be confirmed by a skilled provider. In our study, severity levels were used as a proxy for MHC needs, revealing a graded association between distress levels and MHC use: 11% of those with no distress, 23% with moderate distress, and 47% with severe distress used services during the 6-month follow-up.

In addition, we lacked data on privately funded care, which could explain the lower utilization by affluent persons. However, prescribed drug records captured some utilization and exclusive use of privately funded care in Sweden is likely low (OECD, 2023).

## Conclusion

Despite generally equitable MHC use across education and income groups with higher MHC needs, low-educated individuals faced inequities in primary care and underutilized non-physician services. Interventions are needed to improve MHC access for underserved groups with unmet need.

## Supporting information

Muwonge et al. supplementary material Muwonge et al. supplementary material

## Data Availability

Request SPHC data: Hälsa Stockholm - för forskare (regionstockholm.se)

## Supplementary material

The supplementary material for this article can be found at https://doi.org/10.1017/S2045796024000842.
