# Supplementary material for: Exploring socio-economic inequalities in mental healthcare utilization in adults with self-reported psychological distress: a survey-registry linked cohort design
Source: Epidemiol Psychiatr Sci. 2025 Jan 23;34:e6. doi: 10.1017/S2045796024000842 (PMC11822448; doi:10.1017/S2045796024000842)
Supplement: Muwonge et al. supplementary material [file S2045796024000842sup001.docx]

**Title: Exploring socioeconomic inequalities in mental healthcare utilization in adults with self-reported psychological distress – a survey-registry linked cohort design**

J.J. Muwonge^1,2^ (ORCID: 0000-0002-9219-9752), C. Dalman^1,2^ (ORCID: 0000-0002-3579-2357), B. Burström^1,2^ (ORCID: [0000-0001-5770-9422](https://orcid.org/0000-0001-5770-9422)), B. Jablonska^1,2^ (ORCID: 0000-0002-0246-6643), A-C. Hollander^1^ (ORCID:[0000-0002-1246-5804](https://orcid.org/0000-0002-1246-5804))

1. Department of Global Public Health, Karolinska Institute, Stockholm, Sweden
2. Centre for Epidemiology and Community Medicine, Stockholm, Sweden

Corresponding author

Name: Joseph Jr. Muwonge

Contact: [joseph.junior.muwonge@ki.se](mailto:joseph.junior.muwonge@ki.se)

**Methods**

More details concerning the outcome variable

- Psychiatric diagnoses were based on the tenth revision of the International Classification of Diseases (ICD-10) diagnostic codes: F00-F99, X60-X84, Z72820, Z915, G47, R45851, and T1491.
- Psychotropic medication was identified using the following Anatomical Therapeutic Chemical code (ATC) codes: N05A, N05B, N05C, N06A, N06B, N07BB, and N07BC
- Psychosocial support in primary and secondary outpatient care was identified using the following codes: Uppdragtyp (301, 320), KLIN (950, 951, 955), and VDG1-5 (74, 75, 96; meeting with a psychologist, counsellor, and psychotherapist).
- Visits where they met a doctor were retrieved using the codes 1-69 from the VDG1-5 columns and visits where they met a psychologist, counsellor or psychotherapist were retrieved using the codes 74, 75, & 96 from the VDG1-5 columns in the outpatient register.
- Healthcare level (Vårdnivå: vardniva) was identified using the codes: ‘01’ for primary care and ‘02’ for secondary care.
- Visit type (besökstyp: btyp) was identified using the following codes: btyp in ('6', '9', 'U', 'W', 'Y') for digital visits and btyp in ('0','1','2','8','A', 'B', 'D', 'E', 'F', 'G', 'H', 'K', 'L', 'M', 'N', 'P') for physical visits.

Pooling procedures of the 2014 and 2021 survey waves

The main reason to pool surveys was to increase our sample size and statistical power to identify small subgroup differences e.g., across a combination of sex + age-groups + disorder type or healthcare level. In addition, the enhanced statistical power made it possible to investigate the interaction effects of psychological distress on the relationship between SEP and the utilization of MHC. The 2014 and 2021 survey waves were pooled because they were more recent and since they had optimal coverage of primary care records. Pooling was possible since the Stockholm public surveys have used similar sampling procedures and have measured similar constructs over time. In addition, the distribution of the SEP indicators in the two survey years, was mostly comparable. However, there were some differences across waves. In 2021, more individuals used MHC compared to 2014. This makes sense because utilization of MHC has generally increased over time [28]. However, the most significant difference was in the utilization of primary care. We suspect that in 2014, there might have been fewer primary care facilities reporting to VAL-databases since reporting of primary care records only started in 2013. Furthermore, in 2014, 39 suburbs and municipalities in Stockholm County were used in the stratified sampling while 38 were used in 2021. Two surburbs, Hägersten-Liljeholmen and Älvsjö, were joined into one, Hägersten-Älvsjö in 2020 [29]. Strata were kept unchanged during the pooling, thus Älvsjö stratum is only present for the 2014 wave.

Due to the use of different identification systems in 2014 and 2021, it was impossible to detect potential overlap or repeated observations between the two waves. Nevertheless, we consider these populations to be largely independent. The likelihood of any overlap occurring between two separate cross-sectional surveys conducted seven years apart is minimal.

After pooling the datasets, calibrated weights were divided by the number of waves (2) before using them in the analyses. The readjustment of weights was done to ensure a balanced target population size, while also retaining between-wave differences in the composition of the target population e.g., the proportion of migrants in Sweden was higher in 2021.

Syntax for the two research questions

| **STATA syntax**   1. Adding surveyset parameters to get survey-design adjusted standard errors   svyset [pweight = weight], strata(strata_variable)   1. *Research question 1*     1. *logistic regression predicting gained MHC access; estimates are presented within each distress stratum*   svy:logistic anyuse6M i.distress ib3.education_stat#i.distress age i.sex i.migration_stat i.survey_wave, base   - 1. *P-values from Wald tests; to show if estimates are significantly different across distress strata*   test 1. education_stat#1.distress=1. education_stat#2.distress=1. education_stat#3.distress  test 2. education_stat#1.distress=2. education_stat#2.distress=2. education_stat#3.distress   1. *Research question 2*     1. *negative binomial regression predicting frequency of visits among MHC users; estimates are presented within each distress stratum*   svy:nbreg N_visits6M i.distress ib3.education_stat#i.distress age i.sex i.migration_stat i.survey_wave if anyuse6M ==1, base   - 1. *estimating rate ratios*   nbreg, irr   - 1. *p-values from Wald tests; to show if estimates are significantly different across distress strata*   test 1. education_stat#1.distress=1. education_stat#2.distress=1. education_stat#3.distress  test 2. education_stat#1.distress=2. education_stat#2.distress=2. education_stat#3.distress |
| --- |

**Supplementary results**

2021 survey wave

N= 23,066

2014 survey wave

N= 22,250

Total number of participants in pooled sample

N=45,316

**Excluded:**

- Aged 16-17 years or 65+, n= 13,544
- Died or emigrated during follow-up:
  - 2014 = 11 died & 113 emigrated
  - 2021 = 3 died & 88 emigrated

**Sample used in analysis:**

N = 31,433

18-64 years

*Figure S1. Derivation of the analytic sample.*

**Table S1. Moderated association between SEP and MHC use at least once within 6 months after survey-response.** *ORs (95% CIs) shown for Total sample.*

|  | **No distress** | | **Moderate** | | **Severe** | | **Wald test, p-value** |
| --- | --- | --- | --- | --- | --- | --- | --- |
|  | **Model1** | **Model2** | **Model1** | **Model2** | **Model1** | **Model2** |  |
| **SEP** | **ORs (95% CI)** | **ORs (95% CI)** | **ORs (95% CI)** | **ORs (95% CI)** | **ORs (95% CI)** | **ORs (95% CI)** |  |
| **Education level** |  |  |  |  |  |  |  |
| Primary | **1.50 (1.24, 1.81)** | **1.74 (1.43, 2.12)** | **1.31 (1.10,  1.56)** | **1.53 (1.28, 1.84)** | 1.19 (0.90, 1.57) | 1.30 (0.98, 1.72)* | 0.2414 |
| Secondary | 1.06 (0.94, 1.20) | 1.06 (0.94, 1.20) | 1.00 (0.90, 1.12) | 1.00 (0.89, 1.12) | 1.19 (0.98, 1.46) | 1.20 (0.98, 1.47)* | 0.2917 |
| Post-secondary (ref.) | 1 | 1 | 1 | 1 | 1 | 1 |  |
| **Household income** |  |  |  |  |  |  |  |
| Low | **1.42 (1.24, 1.63)** | **1.92 (1.66, 2.21)** | **1.35 (1.18, 1.54)** | **1.68 (1.47, 1.92)** | **1.82 (1.43, 2.32)** | **2.10 (1.64, 2.69)** | 0.1914 |
| Middle | 0.99 (0.87, 1.13) | 1.11 (0.97, 1.27)* | 1.08 (0.95, 1.24)* | **1.16 (1.01, 1.33)** | **1.45 (1.12, 1.88)** | **1.57 (1.20, 2.05)** | 0.0697 |
| High (ref.) | 1 | 1 | 1 | 1 | 1 | 1 |  |
|  | - Model1 – crude interaction model, Model2 – adjusted for wave effects, sex, age (continuous), and migration status. - Wald test – tests whether estimates in no distress = estimates in moderate distress = estimates in Severe distress. Only done for Model 2 estimates. - Reference categories – post-secondary education & highest income tertile.   *borderline significant | | | | | | |

**Table S2. Moderated association between SEP and MHC use at least once within 6 months after survey-response.** *ORs (95% CIs) shown separately for men and women.*

|  | **Men** | | | | | | | **Women** | | | | | | |
| --- | --- | --- | --- | --- | --- | --- | --- | --- | --- | --- | --- | --- | --- | --- |
|  | **No distress** | | **Moderate** | | **Severe** | |  | **No distress** | | **Moderate** | | **Severe** | |  |
|  | **Model1** | **Model2** | **Model1** | **Model2** | **Model1** | **Model2** |  | **Model1** | **Model2** | **Model1** | **Model2** | **Model1** | **Model2** |  |
| **SEP** | **ORs (95% CI)** | **ORs (95% CI)** | **ORs (95% CI)** | **ORs (95% CI)** | **ORs (95% CI)** | **ORs (95% CI)** | **Wald test, p-value** | **ORs (95% CI)** | **ORs (95% CI)** | **ORs (95% CI)** | **ORs (95% CI)** | **ORs (95% CI)** | **ORs (95% CI)** | **Wald test, p-value** |
| **Education level** |  |  |  |  |  |  |  |  |  |  |  |  |  |  |
| Primary | **2.05 (1.56, 2.68)** | **2.23 (1.69, 2.93)** | **1.70 (1.29, 2.24)** | **1.88 (1.42, 2.48)** | **1.62 (1.02, 2.57)** | **1.69 (1.06, 2.69)** | 0.5172 | 1.24 (0.94, 1.62) | 1.32 (1.00, 1.74)* | 1.15 (0.92, 1.45) | **1.31 (1.03, 1.65)** | 1.05 (0.74, 1.50) | 1.12 (0.78, 1.60) | 0.7363 |
| Secondary | 1.07 (0.88, 1.31) | 1.05 (0.86, 1.28) | 1.04 (0.85, 1.27) | 1.02 (0.84, 1.25) | **1.71 (1.20, 2.43)** | **1.74 (1.21, 2.49)** | 0.0310 | 1.12 (0.96, 1.30) | 1.08 (0.93, 1.26) | 1.00 (0.87, 1.15) | 0.99 (0.86, 1.13) | 1.01 (0.79, 1.29) | 0.97 (0.76, 1.24) | 0.6063 |
| Post-secondary (ref.) | 1 | 1 | 1 | 1 | 1 | 1 |  | 1 | 1 | 1 | 1 | 1 | 1 |  |
| **Household income** |  |  |  |  |  |  |  |  |  |  |  |  |  |  |
| Low | **1.45 (1.17, 1.81)** | **1.97 (1.57, 2.48)** | **1.39 (1.11, 1.74)** | **1.80 (1.42, 2.27)** | 1.41 (0.94, 2.14) | **1.65 (1.09, 2.51)** | **0.7053** | **1.34 (1.13, 1.59)** | **1.88 (1.56, 2.25)** | **1.30 (1.10, 1.52)** | **1.61 (1.36, 1.90)** | **2.08 (1.54, 2.81)** | **2.43 (1.79, 3.31)** | **0.0571** |
| Middle | 0.99 (0.80, 1.22) | 1.12 (0.90, 1.39) | 1.17 (0.93, 1.47) | **1.29 (1.03, 1.62)** | 1.29 (0.83, 2.00) | 1.42 (0.91, 2.22) | 0.5203 | 0.98 (0.83, 1.16) | 1.10 (0.93, 1.31) | 1.02 (0.87, 1.20) | 1.09 (0.92, 1.28) | **1.58 (1.15, 2.17)** | **1.67 (1.21, 2.32)** | **0.0542** |
| High (ref.) | 1 | 1 | 1 | 1 | 1 | 1 |  | 1 | 1 | 1 | 1 | 1 | 1 |  |
|  | - Model1 – crude interaction model, Model2 – adjusted for wave effects, age (continuous), and migration status. - Wald test – tests whether estimates in no distress = estimates in moderate distress = estimates in Severe distress. Only done for Model 2 estimates. - Reference categories – post-secondary education & highest income tertile.   *borderline significant | | | | | | | | | | | | | |

**Table S3. Age-stratified moderated association between SEP and MHC use at least once within 6 months after survey-response.** *ORs (95% CIs) shown for Total sample.*

|  | **18 - 29** | | | | | | | **30-64** | | | | | | |
| --- | --- | --- | --- | --- | --- | --- | --- | --- | --- | --- | --- | --- | --- | --- |
|  | **No distress** | | **Moderate** | | **Severe** | |  | **No distress** | | **Moderate** | | **Severe** | |  |
|  | **Model1** | **Model2** | **Model1** | **Model2** | **Model1** | **Model2** | **Wald test, p** | **Model1** | **Model2** | **Model1** | **Model2** | **Model1** | **Model2** | **Wald test, p** |
| **SEP** | **ORs (95% CI)** | **ORs (95% CI)** | **ORs (95% CI)** | **ORs (95% CI)** | **ORs (95% CI)** | **ORs (95% CI)** |  | **ORs (95% CI)** | **ORs (95% CI)** | **ORs (95% CI)** | **ORs (95% CI)** | **ORs (95% CI)** | **ORs (95% CI)** |  |
| **Education level** |  |  |  |  |  |  |  |  |  |  |  |  |  |  |
| Primary | **1.80 (1.16, 2.79)** | **2.69 (1.62, 4.46)** | 1.27 (0.91, 1.76) | **1.71 (1.17, 2.49)** | 1.50 (0.94 , 2.41) | **1.99 (1.19, 3.34)** | 0.2798 | **1.45 (1.17, 1.80)** | **1.51 (1.22, 1.88)** | **1.55 (1.25, 1.93)** | **1.66 (1.32, 2.08)** | 1.10 (0.77, 1.56) | 1.12 (0.79, 1.60) | 0.1876 |
| Secondary | 0.91 (0.62, 1.34) | 1.09 (0.73, 1.63) | 0.91 (0.70, 1.18) | 1.05 (0.80, 1.39) | 1.37 (0.93, 2.02) | **1.65 (1.10, 2.47)** | 0.1678 | 1.10 (0.97, 1.25) | 1.07 (0.94, 1.21) | 1.09 (0.96, 1.24) | 1.02 (0.89, 1.16) | 1.17 (0.92, 1.47) | 1.07 (0.84, 1.36) | 0.8404 |
| Post-secondary (ref.) | 1 | 1 | 1 | 1 | 1 | 1 |  | 1 | 1 | 1 | 1 | 1 | 1 |  |
| **Household income** |  |  |  |  |  |  |  |  |  |  |  |  |  |  |
| Low | 1.16 (0.77, 1.74) | 1.24 (0.82, 1.88) | **1.51 (1.10, 2.09)** | **1.66 (1.20, 2.28)** | **1.85 (1.12, 3.04)** | **2.03 (1.22, 3.37)** | 0.3111 | **1.50 (1.30, 1.73)** | **1.98 (1.70, 2.30)** | **1.41 (1.22, 1.63)** | **1.75 (1.51, 2.04)** | **1.86 (1.41, 2.45)** | **2.17 (1.63, 2.89)** | 0.3109 |
| Middle | 0.79 (0.51, 1.22) | 0.78 (0.50, 1.22) | 1.10 (0.77, 1.55) | 1.11 (0.78, 1.57) | 1.52 (0.90, 2.57) | 1.58 (0.93, 2.69) | 0.1295 | 1.03 (0.90, 1.18) | 1.15 (1.00, 1.31)* | 1.12 (0.97, 1.29)* | **1.20 (1.04, 1.39)** | **1.46 (1.09, 1.96)** | **1.56 (1.15, 2.12)** | 0.2004 |
| High (ref.) | 1 | 1 | 1 | 1 | 1 | 1 |  | 1 | 1 | 1 | 1 | 1 | 1 |  |
|  | - Model1 – crude interaction model, Model2 – adjusted for wave effects, age (continuous), and migration status. - Wald test – tests whether estimates in no distress = estimates in moderate distress = estimates in Severe distress. Only done for Model 2 estimates. - Reference categories – post-secondary education & highest income tertile. - *borderline significant | | | | | | | | | | | | | |


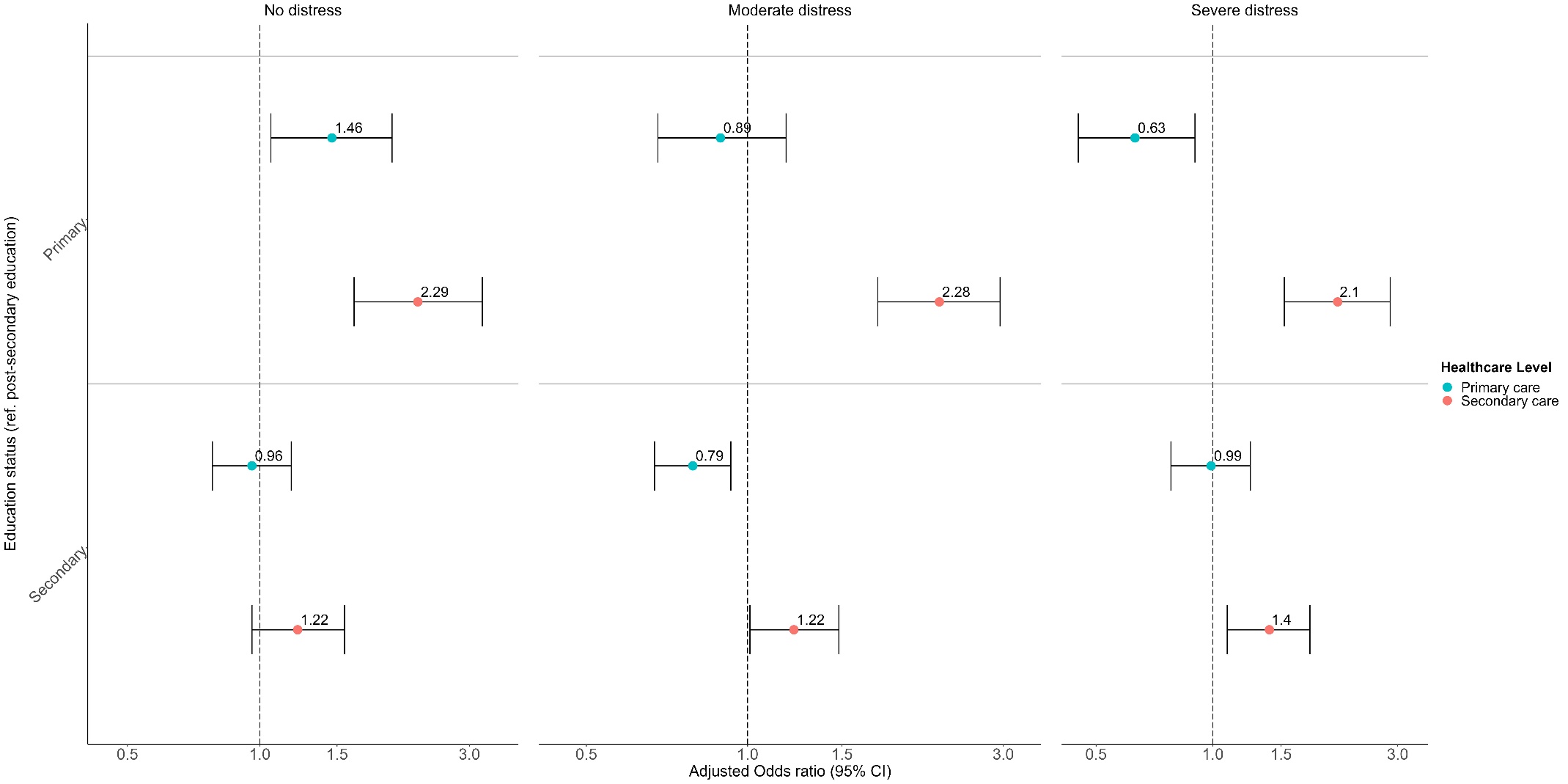


*Figure S2. Odds ratios (log scale) for the moderated association between education status and any MHC use at least once (by healthcare level) within 6 months after survey response. Models were adjusted for wave effects, sex, age (continuous), and migration status.*

**Table S4. Moderated association between SEP and MHC use at least once (by healthcare level) within 6 months after survey-response.** *ORs (95% CIs) shown for Total sample.*

|  | **Primary care** | | | | | | | **Secondary care** | | | | | | |
| --- | --- | --- | --- | --- | --- | --- | --- | --- | --- | --- | --- | --- | --- | --- |
|  | **No distress** | | **Moderate** | | **Severe** | |  | **No distress** | | **Moderate** | | **Severe** | |  |
|  | **Model1** | **Model2** | **Model1** | **Model2** | **Model1** | **Model2** | **Wald test, p** | **Model1** | **Model2** | **Model1** | **Model2** | **Model1** | **Model2** | **Wald test, p** |
| **SEP** | **ORs (95% CI)** | **ORs (95% CI)** | **ORs (95% CI)** | **ORs (95% CI)** | **ORs (95% CI)** | **ORs (95% CI)** |  | **ORs (95% CI)** | **ORs (95% CI)** | **ORs (95% CI)** | **ORs (95% CI)** | **ORs (95% CI)** | **ORs (95% CI)** |  |
| **Education level** |  |  |  |  |  |  |  |  |  |  |  |  |  |  |
| Primary | 1.23 (0.90, 1.69) | **1.46 (1.06, 2.00)** | 0.79 (0.60, 1.03)* | 0.89 (0.68, 1.18) | **0.63 (0.45, 0.89)** | **0.63 (0.45, 0.90)** | 0.0021 | **2.13 (1.52, 2.98)** | **2.29 (1.64, 3.21)** | **2.16 (1.67, 2.80)** | **2.28 (1.75, 2.96)** | **1.99 (1.45, 2.72)** | **2.10 (1.53, 2.87)** | 0.9050 |
| Secondary | 0.92 (0.75, 1.13) | 0.96 (0.78, 1.18) | **0.78 (0.66, 0.92)** | **0.79 (0.67, 0.93)** | 0.98 (0.77, 1.23) | 0.99 (0.78, 1.25) | 0.2116 | 1.20 (0.94, 1.53) | 1.22 (0.96, 1.56)* | **1.22 (1.01, 1.47)** | **1.22 (1.01, 1.48)** | **1.39 (1.09, 1.78)** | **1.40 (1.09, 1.78)** | 0.6682 |
| Post-secondary (ref.) | 1 | 1 | 1 | 1 | 1 | 1 |  | 1 | 1 | 1 | 1 | 1 | 1 |  |
| **Household income** |  |  |  |  |  |  |  |  |  |  |  |  |  |  |
| Low | **1.36 (1.09, 1.72)** | **1.66 (1.31, 2.11)** | **1.30 (1.08, 1.57)** | **1.51 (1.24, 1.83)** | **1.38 (1.04, 1.84)** | **1.43 (1.07, 1.91)** | 0.6900 | **2.98 (2.24, 3.97)** | **3.51 (2.62, 4.70)** | **2.47 (1.96, 3.12)** | **2.81 (2.22, 3.56)** | **3.12 (2.22, 4.39)** | **3.56 (2.51, 5.04)** | 0.3794 |
| Middle | 1.02 (0.82, 1.28) | 1.10 (0.88, 1.38) | 1.18 (0.97, 1.42)* | 1.21 (1.00, 1.47)* | 1.35 (0.99, 1.83)* | 1.35 (0.99, 1.84)* | 0.5610 | **1.44 (1.07, 1.96)** | **1.49 (1.10, 2.02)** | 1.29 (1.00, 1.66)* | **1.32 (1.02, 1.70)** | **2.08 (1.44, 3.00)** | **2.13 (1.47, 3.09)** | 0.1091 |
| High (ref.) | 1 | 1 | 1 | 1 | 1 | 1 |  | 1 | 1 | 1 | 1 | 1 | 1 |  |
|  | - Model1 – crude interaction model, Model2 – adjusted for wave effects, age (continuous), and migration status. - Wald test – tests whether estimates in no distress = estimates in moderate distress = estimates in Severe distress. Only done for Model 2 estimates. - Reference categories – post-secondary education & highest income tertile. - *borderline significant | | | | | | | | | | | | | |


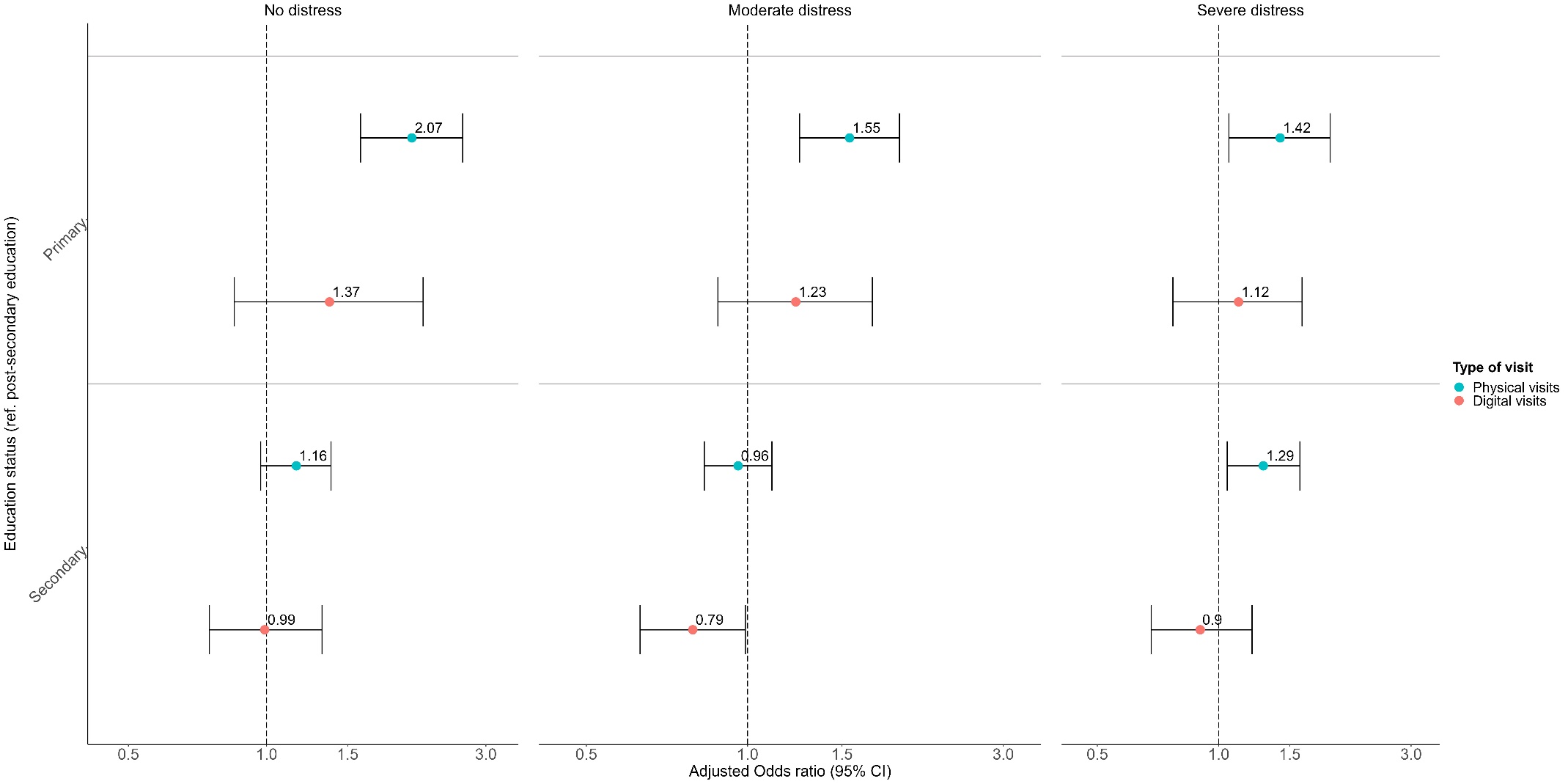


*Figure S3. Odds ratios (log scale) for the moderated association between education status and any MHC use at least once (by type of visit) within 6 months after survey response. Models were adjusted for wave effects, sex, age (continuous), and migration status.*

**Table S5. Moderated association between SEP and MHC use at least once (by visit type) within 6 months after survey-response.** *ORs (95% CIs) shown for Total sample.*

|  | **Physical visits** | | | | | | | **Digital visits** | | | | | | |
| --- | --- | --- | --- | --- | --- | --- | --- | --- | --- | --- | --- | --- | --- | --- |
|  | **No distress** | | **Moderate** | | **Severe** | |  | **No distress** | | **Moderate** | | **Severe** | |  |
|  | **Model1** | **Model2** | **Model1** | **Model2** | **Model1** | **Model2** | **Wald test, p** | **Model1** | **Model2** | **Model1** | **Model2** | **Model1** | **Model2** | **Wald test, p** |
| SEP | **ORs (95% CI)** | **ORs (95% CI)** | **ORs (95% CI)** | **ORs (95% CI)** | **ORs (95% CI)** | **ORs (95% CI)** |  | **ORs (95% CI)** | **ORs (95% CI)** | **ORs (95% CI)** | **ORs (95% CI)** | **ORs (95% CI)** | **ORs (95% CI)** |  |
| **Education level** |  |  |  |  |  |  |  |  |  |  |  |  |  |  |
| Primary | **1.86 (1.45, 2.40)** | **2.07 (1.60, 2.67)** | **1.42 (1.15, 1.76)** | **1.55 (1.25, 1.92)** | 1.32 (1.00, 1.76)* | **1.42 (1.06, 1.89)** | 0.1104 | 1.07 (0.67, 1.70) | 1.37 (0.85, 2.19) | 1.08 (0.78, 1.50) | 1.23 (0.88, 1.71) | 1.31 (0.93, 1.86) | 1.12 (0.77, 1.61) | 0.7998 |
| Secondary | 1.13 (0.95, 1.35)* | 1.16 (0.97, 1.38)* | 0.96 (0.83, 1.10) | 0.96 (0.83, 1.11) | **1.28 (1.04, 1.57)** | **1.29 (1.05, 1.59)** | 0.0542 | 0.88 (0.66, 1.16) | 0.99 (0.75, 1.32) | **0.75 (0.60, 0.93)** | **0.79 (0.63, 0.99)** | 0.91 (0.70, 1.20) | 0.90 (0.68, 1.21) | 0.4374 |
| Post-secondary (ref.) | 1 | 1 | 1 | 1 | 1 | 1 |  | 1 | 1 | 1 | 1 | 1 | 1 |  |
| **Household income** |  |  |  |  |  |  |  |  |  |  |  |  |  |  |
| Low | **2.13 (1.74, 2.60)** | **2.51 (2.04, 3.09)** | **1.86 (1.57, 2.20)** | **2.10 (1.77, 2.50)** | **2.46 (1.89, 3.20)** | **2.74 (2.10, 3.58)** | 0.1878 | **1.81 (1.32, 2.50)** | **2.24 (1.61, 3.11)** | **1.44 (1.11, 1.86)** | **1.72 (1.32, 2.24)** | **1.73 (1.22, 2.46)** | **1.64 (1.13, 2.39)** | 0.3646 |
| Middle | 1.19 (0.97, 1.47)* | **1.25 (1.02, 1.54)** | **1.22 (1.03, 1.46)** | **1.26 (1.06, 1.50)** | **1.77 (1.34, 2.35)** | **1.85 (1.39, 2.46)** | 0.0577 | 1.12 (0.81, 1.55) | 1.16 (0.83, 1.61) | 1.22 (0.94, 1.58) | 1.19 (0.91, 1.55) | **1.66 (1.15, 2.41)** | 1.48 (1.00, 2.18)* | 0.6008 |
| High (ref.) | 1 | 1 | 1 | 1 | 1 | 1 |  | 1 | 1 | 1 | 1 | 1 | 1 |  |
|  | - Model1 – crude interaction model, Model2 – adjusted for wave effects, age (continuous), and migration status. - Wald test – tests whether estimates in no distress = estimates in moderate distress = estimates in Severe distress. Only done for Model 2 estimates. - Reference categories – post-secondary education & highest income tertile. - *borderline significant | | | | | | | | | | | | | |

**Table S6. Moderated association between SEP and incident MHC use within 6 months after survey-response.** *ORs (95% CIs) shown for Total sample excluding individuals who used MHC services six months prior to survey response.*

|  | **No distress** | | **Moderate** | | **Severe** | | **Wald test, p-value** |
| --- | --- | --- | --- | --- | --- | --- | --- |
|  | **Model1** | **Model2** | **Model1** | **Model2** | **Model1** | **Model2** |  |
| **SEP** | **ORs (95% CI)** | **ORs (95% CI)** | **ORs (95% CI)** | **ORs (95% CI)** | **ORs (95% CI)** | **ORs (95% CI)** |  |
| **Education level** |  |  |  |  |  |  |  |
| Primary | 1.33 (0.96, 1.84)* | **1.49 (1.07, 2.07)** | 1.07 (0.77, 1.48) | 1.20 (0.86, 1.68) | 0.82 (0.49, 1.40) | 0.87 (0.51, 1.47) | 0.2249 |
| Secondary | 0.97 (0.79, 1.20) | 0.99 (0.80, 1.22) | **0.76 (0.62, 0.94)** | **0.76 (0.62, 0.94)** | 0.92 (0.64, 1.33) | 0.92 (0.64, 1.32) | 0.2311 |
| Post-secondary (ref.) | 1 | 1 | 1 | 1 | 1 | 1 |  |
| **Household income** |  |  |  |  |  |  |  |
| Low | 1.12 (0.88, 1.41) | **1.33 (1.04, 1.71)** | 0.90 (0.71, 1.14) | 1.03 (0.81, 1.31) | **1.83 (1.16, 2.89)** | **2.05 (1.3, 3.24)** | 0.0238 |
| Middle | 0.80 (0.64, 1.01)* | 0.86 (0.68, 1.08) | 0.91 (0.73, 1.15) | 0.95 (0.76, 1.20) | **1.64 (1.03, 2.62)** | **1.72 (1.08, 2.74)** | 0.0309 |
| High (ref.) | 1 | 1 | 1 | 1 | 1 | 1 |  |
|  | - Model1 – crude interaction model, Model2 – adjusted for wave effects, sex, age (continuous), and migration status. - Wald test – tests whether estimates in no distress = estimates in moderate distress = estimates in Severe distress. Only done for Model 2 estimates. - Reference categories – post-secondary education and highest income tertile.   *borderline significant | | | | | | |

**Table S7. Moderated association between SEP and the frequency of outpatient visits within 6 months after survey-response among MHC users.** *Rate Ratios, RRs (95% CIs) shown for Total sample.*

|  | **No distress** | | **Moderate distress** | | **Severe distress** | | **Wald test, p-value** |
| --- | --- | --- | --- | --- | --- | --- | --- |
|  | **Model 1** | **Model 2** | **Model 1** | **Model 2** | **Model 1** | **Model 2** |  |
|  | ***RR (95% CI)*** | ***RR (95% CI)*** | ***RR (95% CI)*** | ***RR (95% CI)*** | ***RR (95% CI)*** | ***RR (95% CI)*** |  |
| **Education level** |  |  |  |  |  |  |  |
| Primary | 1.34 (0.96, 1.86)* | 1.24 (0.90, 1.72) | 1.09 (0.82, 1.45) | 1.06 (0.76, 1.47) | 0.89 (0.72, 1.09) | 0.82 (0.67, 1.00)* | 0.0771 |
| Secondary | **1.43 (1.08, 1.90)** | **1.47 (1.14, 1.88)** | 0.96 (0.81, 1.13) | 0.96 (0.82, 1.13) | 0.95 (0.79, 1.14) | 0.99 (0.82, 1.19) | 0.0170 |
| Post-secondary (ref.) | 1 | 1 | 1 | 1 | 1 | 1 |  |
| **Household income** |  |  |  |  |  |  |  |
| Low | **1.89 (1.41, 2.53)** | **1.82 (1.39, 2.40)** | **1.46 (1.20, 1.76)** | **1.40 (1.15, 1.70)** | **1.33 (1.04, 1.69)** | 1.21 (0.94, 1.56) | 0.0965 |
| Middle | 1.28 (0.97, 1.68)* | 1.23 (0.95, 1.60)* | 1.20 (0.98, 1.47)* | 1.17 (0.95, 1.43)* | 1.24 (0.96, 1.60)* | 1.10 (0.85, 1.43) | 0.8289 |
| High (ref.) | 1 | 1 | 1 | 1 | 1 | 1 |  |
|  | - Model1 – crude interaction model, Model2 – adjusted for wave effects, sex, age (continuous), and migration status. - Wald test – tests whether estimates in no distress = estimates in moderate distress = estimates in Severe distress. Only done for Model 2 estimates. - Reference categories – post-secondary education and highest income tertile. - *borderline significant | | | | | | |

**Table S8. Sex-stratified moderated association between SEP and the frequency of visits within 6 months after survey-response among MHC users.** *Rate Ratios, RR (95% CIs) shown.*

|  | **Men** | | | | | | | **Women** | | | | | | |
| --- | --- | --- | --- | --- | --- | --- | --- | --- | --- | --- | --- | --- | --- | --- |
|  | **No distress** | | **Moderate** | | **Severe** | |  | **No distress** | | **Moderate** | | **Severe** | |  |
|  | **Model 1** | **Model 2** | **Model 1** | **Model 2** | **Model 1** | **Model 2** |  | **Model 1** | **Model 2** | **Model 1** | **Model 2** | **Model 1** | **Model 2** |  |
|  | **RR (95% CI)** | **RR (95% CI)** | **RR (95% CI)** | **RR (95% CI)** | **RR (95% CI)** | **RR (95% CI)** | **Wald test, p** | **RR (95% CI)** | **RR (95% CI)** | **RR (95% CI)** | **RR (95% CI)** | **RR (95% CI)** | **RR (95% CI)** | **Wald test, p** |
| **Education level** |  |  |  |  |  |  |  |  |  |  |  |  |  |  |
| Primary | 1.12 (0.71, 1.79) | 0.95 (0.63, 1.42) | 1.23 (0.73, 2.07) | 1.17 (0.65, 2.12) | 0.95 (0.66 - 1.38) | 0.97 (0.67 - 1.41) | 0.8211 | **1.63 (1.02, 2.62)** | **1.65 (1.03, 2.64)** | 1.04 (0.76, 1.43) | 1.02 (0.73, 1.42) | 0.89 (0.69, 1.14) | 0.79 (0.62, 1.00)* | 0.0210 |
| Secondary | 1.87 (1.16, 3.02) | 1.76 (1.16, 2.68) | 1.21 (0.87, 1.67) | 1.23 (0.90, 1.69) | 1.15 (0.83, 1.60) | 1.11 (0.81, 1.52) | 0.2147 | 1.14 (0.87, 1.50) | 1.24 (0.95, 1.62) | 0.86 (0.71, 1.04)* | 0.86 (0.72, 1.04)* | 0.89 (0.70, 1.11) | 0.94 (0.75, 1.19) | 0.0899 |
| Post-secondary (ref.) | 1 | 1 | 1 | 1 | 1 | 1 |  | 1 | 1 | 1 | 1 | 1 | 1 |  |
| **Household income** |  |  |  |  |  |  |  |  |  |  |  |  |  |  |
| Low | **2.21 (1.35, 3.60)** | **2.12 (1.36, 3.32)** | 1.34 (0.93, 1.93) | 1.34 (0.92, 1.94) | 1.41 (0.95, 2.09) * | 1.39 (0.93, 2.09) | 0.2676 | **1.68 (1.21, 2.32)** | **1.62 (1.17, 2.25)** | **1.52 (1.22, 1.90)** | **1.43 (1.15, 1.78)** | 1.27 (0.93, 1.71) | 1.12 (0.82, 1.53) | 0.2468 |
| Middle | 1.43 (0.93, 2.20) | 1.35 (0.90, 2.03) | 1.11 (0.75, 1.63) | 1.07 (0.73, 1.58) | **1.58 (1.03, 2.41)** | 1.45 (0.94, 2.24) | 0.5517 | 1.17 (0.84, 1.64) | 1.16 (0.84, 1.60) | 1.25 (0.99, 1.59)* | 1.22 (0.97, 1.53)* | 1.09 (0.80, 1.49) | 0.97 (0.70, 1.34) | 0.5255 |
| High (ref.) | 1 | 1 | 1 | 1 | 1 | 1 |  | 1 | 1 | 1 | 1 | 1 | 1 |  |
|  | - Model1 – crude interaction model, Model2 – adjusted for wave effects, sex, age (continuous), and migration status. - Wald test – tests whether estimates in no distress = estimates in moderate distress = estimates in Severe distress. Only done for Model 2 estimates. - Reference categories – post-secondary education and highest income tertile. - *borderline significant | | | | | | | | | | | | | |

**Table S9. Age-stratified moderated association between SEP and the frequency of visits within 6 months after survey-response among MHC users.** *Rate Ratios, RR (95% CIs) shown.*

|  | **18, 29** | | | | | | | **30-64** | | | | | | |
| --- | --- | --- | --- | --- | --- | --- | --- | --- | --- | --- | --- | --- | --- | --- |
|  | **No distress** | | **Moderate** | | **Severe** | |  | **No distress** | | **Moderate** | | **Severe** | |  |
|  | **Model1** | **Model2** | **Model1** | **Model2** | **Model1** | **Model2** | **Wald test, p** | **Model1** | **Model2** | **Model1** | **Model2** | **Model1** | **Model2** | **Wald test, p** |
| **SEP** | **RRs (95% CI)** | **RRs (95% CI)** | **RRs (95% CI)** | **RRs (95% CI)** | **RRs (95% CI)** | **RRs (95% CI)** |  | **RRs (95% CI)** | **RRs (95% CI)** | **RRs (95% CI)** | **RRs (95% CI)** | **RRs (95% CI)** | **RRs (95% CI)** |  |
| **Education level** |  |  |  |  |  |  |  |  |  |  |  |  |  |  |
| Primary | 1.49 (0.87, 2.55) | 1.34 (0.75, 2.41) | 1.26 (0.86, 1.86) | 1.20 (0.80, 1.80) | 0.86 (0.61, 1.19) | 0.78 (0.54, 1.13) | 0.1356 | 1.04 (0.70, 1.55) | 1.22 (0.81, 1.85) | 0.99 (0.65, 1.49) | 1.14 (0.73, 1.78) | 0.80 (0.61, 1.04)* | 0.88 (0.68, 1.14) | 0.3358 |
| Secondary | 1.55 (0.92, 2.61) | 1.48 (0.87, 2.52) | 1.32 (0.96, 1.83)* | 1.32 (0.95, 1.83)* | 0.72 (0.51, 1.00)* | **0.64 (0.46, 0.90)** | 0.0029 | 1.38 (1.00, 1.92)* | **1.47 (1.12, 1.94)** | 0.85 (0.69, 1.03)* | 0.93 (0.77, 1.13) | 1.05 (0.84, 1.31) | 1.16 (0.94, 1.45) | 0.0276 |
| Post-secondary (ref.) | 1 | 1 | 1 | 1 | 1 | 1 |  | 1 | 1 | 1 | 1 | 1 | 1 |  |
| **Household income** |  |  |  |  |  |  |  |  |  |  |  |  |  |  |
| Low | 0.99 (0.58, 1.70) | 1.06 (0.62, 1.82) | 0.99 (0.67, 1.46) | 1.02 (0.70, 1.49) | **1.74 (1.17, 2.60)** | **1.86 (1.25, 2.77)** | 0.0696 | **2.17 (1.55, 3.04)** | **2.04 (1.50, 2.77)** | **1.59 (1.28, 1.97)** | **1.52 (1.21, 1.91)** | 1.16 (0.86, 1.56) | 1.08 (0.81, 1.45) | 0.0145 |
| Middle | 1.07 (0.59, 1.93) | 1.06 (0.59, 1.90) | 0.84 (0.55, 1.27) | 0.89 (0.59, 1.33) | 1.40 (0.92, 2.14) | 1.40 (0.93, 2.13) | 0.3003 | 1.31 (0.98, 1.75)* | 1.24 (0.93, 1.65) | **1.31 (1.04, 1.66)** | 1.24 (0.98, 1.55)* | 1.17 (0.86, 1.59) | 1.05 (0.77, 1.42) | 0.6445 |
| High (ref.) | 1 | 1 | 1 | 1 | 1 | 1 |  | 1 | 1 | 1 | 1 | 1 | 1 | 1 |
|  | - Model1 – crude interaction model, Model2 – adjusted for wave effects, sex, age (continuous), and migration status. - Wald test – tests whether estimates in no distress = estimates in moderate distress = estimates in Severe distress. Only done for Model 2 estimates. - Reference categories – post-secondary education and highest income tertile. - *borderline significant | | | | | | | | | | | | | |


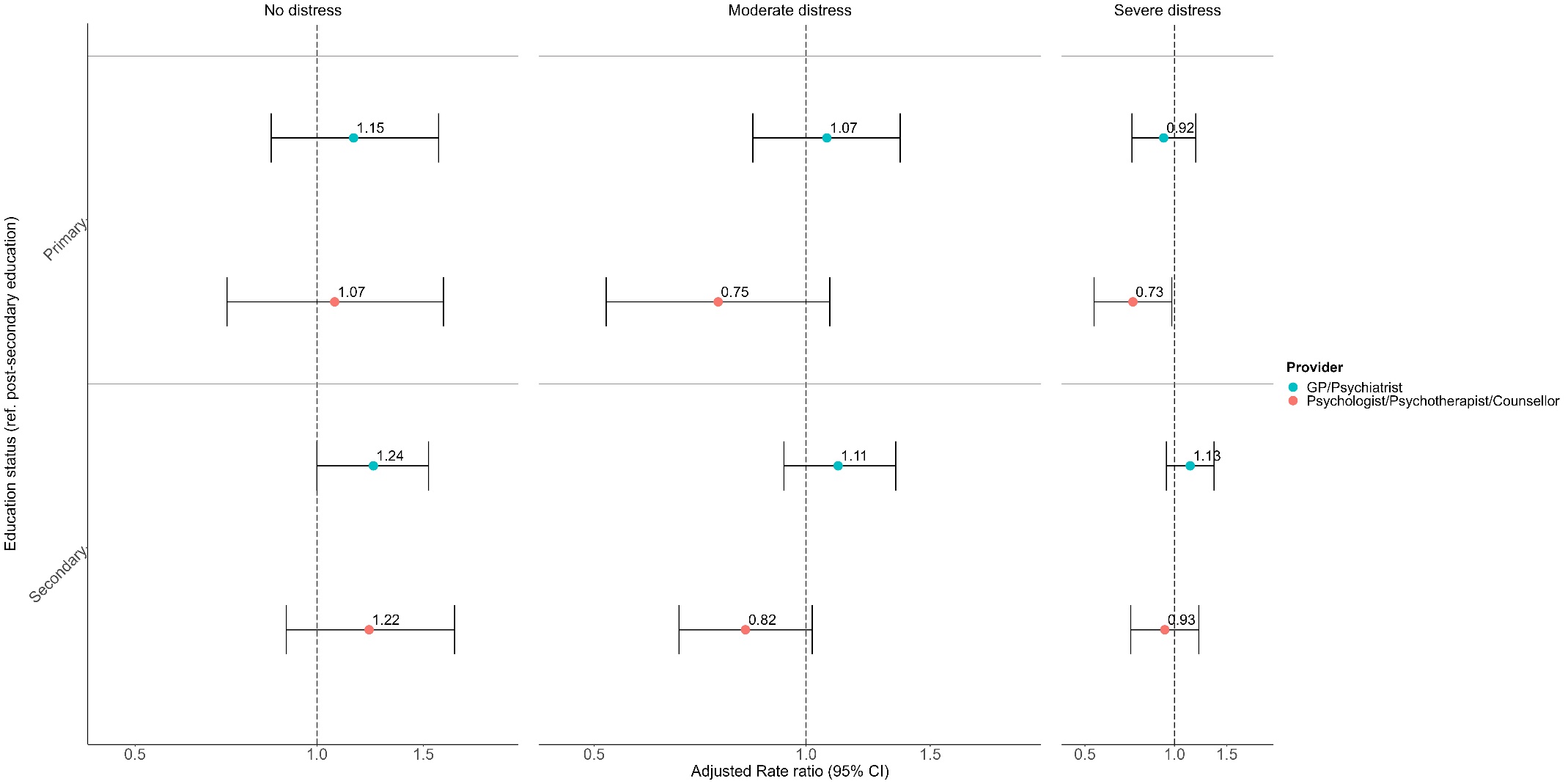


*Figure S7. Rate ratios (log scale) for the moderated association between education status and the frequency of visits (by provider visited) within 6 months after survey response. Models were adjusted for wave effects, sex, age (continuous), and migration status.*

**Table S10. Moderated association between SEP and the frequency of visits (by provider visited) within 6 months after survey-response among MHC users.** *Rate Ratios, RR (95% CIs) shown.*

|  | **GP/Psychiatrist** | | | | | | | **Psychologist, counselor, or psychotherapist** | | | | | | |
| --- | --- | --- | --- | --- | --- | --- | --- | --- | --- | --- | --- | --- | --- | --- |
|  | **No distress** | | **Moderate** | | **Severe** | |  | **No distress** | | **Moderate** | | **Severe** | |  |
|  | **Model1** | **Model2** | **Model1** | **Model2** | **Model1** | **Model2** | **Wald test, p** | **Model1** | **Model2** | **Model1** | **Model2** | **Model1** | **Model2** | **Wald test, p** |
| **SEP** | **RRs (95%CI)** | **RRs (95%CI)** | **RRs (95%CI)** | **RRs (95%CI)** | **RRs (95%CI)** | **RRs (95%CI)** |  | **RRs (95%CI)** | **RRs (95%CI)** | **RRs (95%CI)** | **RRs (95%CI)** | **RRs (95%CI)** | **RRs (95%CI)** |  |
| **Education level** |  |  |  |  |  |  |  |  |  |  |  |  |  |  |
| Primary | 1.20 (0.86, 1.68) | 1.15 (0.84, 1.59) | 1.11 (0.88, 1.40) | 1.07 (0.84, 1.36) | 0.97 (0.75, 1.24) | 0.92 (0.72, 1.18) | 0.5112 | 1.22 (0.74, 1.99) | 1.07 (0.71, 1.62) | 0.81 (0.58, 1.15) | 0.75 (0.52, 1.08) | 0.81 (0.61, 1.08) | **0.73 (0.54, 0.98)** | 0.2846 |
| Secondary | 1.22 (0.98, 1.51)* | 1.24 (1.00, 1.53)* | 1.11 (0.91, 1.34) | 1.11 (0.93, 1.34) | 1.13 (0.93, 1.36) | 1.13 (0.94, 1.36) | 0.7255 | 1.17 (0.83, 1.66) | 1.22 (0.89, 1.69) | 0.82 (0.66, 1.02)* | 0.82 (0.66, 1.02)* | 0.89 (0.69, 1.15) | 0.93 (0.71, 1.21) | 0.1375 |
| Post-secondary (ref.) | 1 | 1 | 1 | 1 | 1 | 1 |  | 1 | 1 | 1 | 1 | 1 | 1 |  |
| **Household income** |  |  |  |  |  |  |  |  |  |  |  |  |  |  |
| Low | **1.66 (1.30, 2.12)** | **1.64 (1.29, 2.09)** | **1.49 (1.23, 1.81)** | **1.48 (1.23, 1.79)** | **1.41 (1.12, 1.77)** | **1.36 (1.08, 1.72)** | 0.5492 | **1.50 (1.02, 2.19)** | 1.32 (0.92, 1.88) | **1.33 (1.05, 1.70)** | 1.21 (0.96, 1.54)* | 1.39 (0.99, 1.95)* | 1.18 (0.83, 1.67) | 0.9002 |
| Middle | 1.10 (0.85, 1.42) | 1.10 (0.85, 1.41) | **1.37 (1.09, 1.73)** | **1.35 (1.08, 1.68)** | **1.29 (1.02, 1.64)** | 1.22 (0.96, 1.57)* | 0.4850 | 1.40 (0.93, 2.08) | 1.35 (0.92, 1.98) | 1.17 (0.90, 1.53) | 1.13 (0.87, 1.46) | 1.32 (0.94, 1.86) | 1.09 (0.77, 1.55) | 0.6853 |
| High (ref.) | 1 | 1 | 1 | 1 | 1 | 1 |  | 1 | 1 | 1 | 1 | 1 | 1 |  |
|  | - Model1 – crude interaction model, Model2 – adjusted for wave effects, sex, age (continuous), and migration status. - Wald test – tests whether estimates in no distress = estimates in moderate distress = estimates in Severe distress. Only done for Model 2 estimates. - Reference categories – post-secondary education and highest income tertile. - *borderline significant | | | | | | | | | | | | | |

**Table S11. Association between SEP and not using services despite scoring ≥ 3 on the GHQ-12 or ≥ 8 on the K6**

|  | **Total^a^** | **Sex** | | **Age group** | |
| --- | --- | --- | --- | --- | --- |
|  |  | **Men^b^** | **Women^b^** | **18-29^a^** | **30-64^a^** |
|  | OR(95% CI) | OR(95% CI) | OR(95% CI) | OR(95% CI) | OR(95% CI) |
| **Education level** |  |  |  |  |  |
| Primary | 1.12 (0.99, 1.27)* | 0.96 (0.79, 1.16) | **1.31 (1.10, 1.55)** | 0.86 (0.67, 1.11) | **1.27 (1.08, 1.49)** |
| Secondary | 1.07 (0.99, 1.15)* | 0.94 (0.84, 1.06) | **1.20 (1.08, 1.32)** | 0.96 (0.81, 1.14) | 1.09 (1.00, 1.20)* |
| Post-secondary (ref.) | 1 | 1 | 1 | 1 |  |
| **Household income** |  |  |  |  |  |
| Low | **1.27 (1.15, 1.40)** | **1.41 (1.22, 1.64)** | **1.14 (1.01, 1.29)** | 1.01 (0.83, 1.21) | **1.39 (1.25, 1.55)** |
| Middle | **1.18 (1.07, 1.29)** | **1.29 (1.13, 1.48)** | 1.07 (0.95, 1.21) | 1.07 (0.89, 1.30) | **1.20 (1.08, 1.33)** |
| High (ref.) | 1 | 1 | 1 | 1 |  |
|  | - a-Adjusted for age (continuous), sex, survey wave, and migration status. - b-Adjusted for age (continuous), survey wave, and migration status. - *-borderline significant | | | | |
